# Supplementary material for: Phonon heat transport in cavity-mediated optomechanical nanoresonators
Source: Nat Commun. 2020 Sep 16;11:4656. doi: 10.1038/s41467-020-18426-4 (PMC7494915; doi:10.1038/s41467-020-18426-4)
Supplement: Supplementary file 1 — Supplementary Information [file 41467_2020_18426_MOESM1_ESM.pdf]

Supplementary Information

Phonon Heat Transport in Cavity-Mediated Optomechanical Nanoresonators

Yang et al.

Supplementary Note 1: Equivalent coupled-mode model

The system of the heat transport between two spatially separated nanomechanical resonators is a two-membrane-in-the-middle cavity optomechanical system, in which each membrane couples to a common cavity mode via the optomechanical interaction. The total Hamiltonian of such a system in the rotating frame of the driving laser frequency can be written as $(\hbar = 1)$ [1-4]

$$\hat{H} = -\Delta\hat{a}^\dagger\hat{a} + \omega_1\hat{b}_1^\dagger\hat{b}_1 + \omega_2\hat{b}_2^\dagger\hat{b}_2 - g_1\hat{a}^\dagger\hat{a}\left(\hat{b}_1^\dagger + \hat{b}_1\right) - g_2\hat{a}^\dagger\hat{a}\left(\hat{b}_2^\dagger + \hat{b}_2\right) + i\varepsilon\left(\hat{a}^\dagger - \hat{a}\right). \tag{1}$$

Here $\hat{a}$, $\hat{b}_1$, and $\hat{b}_2$ are the annihilation operators of the cavity mode and the mechanical oscillators, respectively. $\Delta = \omega_L - \omega_C$ is the frequency detuning between the driving laser and the cavity field. $\omega_{1,2}$ is the intrinsic frequency of the mechanical oscillator. $g_{1,2}$ is the optomechanical coupling strength. $\varepsilon = \sqrt{P\kappa_{in}/\hbar\omega_L}$ is the driving strength, where P is the input laser power, and κ_{in} is the loss of the input cavity mirror.

According to Supplementary Equation (1), the equations of motion can be obtained as follows

$$\frac{d}{dt}\hat{a} = -\left(\frac{\kappa}{2} - i\Delta\right)\hat{a} + ig_1\hat{a}\left(\hat{b}_1^\dagger + \hat{b}_1\right) + ig_2\hat{a}\left(\hat{b}_2^\dagger + \hat{b}_2\right) + \varepsilon + \sqrt{\kappa}\hat{a}_{in}, \tag{2}$$

$$\frac{d}{dt}\hat{b}_1 = -\left(\frac{\gamma_1}{2} + i\omega_1\right)\hat{b}_1 + ig_1\hat{a}^\dagger\hat{a} + \sqrt{\gamma_1}\hat{b}_{1in}, \tag{3}$$

$$\frac{d}{dt}\hat{b}_2 = -\left(\frac{\gamma_2}{2} + i\omega_2\right)\hat{b}_2 + ig_2\hat{a}^\dagger\hat{a} + \sqrt{\gamma_2}\hat{b}_{2in}, \tag{4}$$

where $\hat{a}_{in}$ and $\hat{b}_{1in}$ ($\hat{b}_{2in}$) are the thermal noise operators of the cavity mode and the mechanical modes. κ is the total decay of cavity mode. $\gamma_{1,2}$ is the mechanical damping rate. We can obtain the linearized equations of motion in the frequency domain as

$$-i\omega\delta\hat{a}[\omega] = -\left(\frac{\kappa}{2} - i\Delta\right)\delta\hat{a}[\omega] + ig_1\alpha\left(\hat{b}_1^\dagger[\omega] + \hat{b}_1[\omega]\right) + ig_2\alpha\left(\hat{b}_2^\dagger[\omega] + \hat{b}_2[\omega]\right), \tag{5}$$

$$-i\omega\hat{b}_1[\omega] = -\left(\frac{\gamma_1}{2} + i\omega_1\right)\hat{b}_1[\omega] + ig_1\left(\alpha\delta\hat{a}^\dagger[\omega] + \alpha^*\delta\hat{a}[\omega]\right) + \sqrt{\gamma_1}\hat{b}_{1in}[\omega], \tag{6}$$

$$-i\omega\hat{b}_2[\omega] = -\left(\frac{\gamma_2}{2} + i\omega_2\right)\hat{b}_2[\omega] + ig_2\left(\alpha\delta\hat{a}^\dagger[\omega] + \alpha^*\delta\hat{a}[\omega]\right) + \sqrt{\gamma_2}\hat{b}_{2in}[\omega], \tag{7}$$

where $\delta\hat{a}$ is the cavity mode fluctuation and $\alpha = \langle \hat{a} \rangle = \varepsilon / (\kappa / 2 - i\Delta)$ is the mean intracavity field. We drop the counter-rotating terms $\hat{b}_1^\dagger[\omega]$ and $\hat{b}_2^\dagger[\omega]$, as well as the thermal driving terms $\sqrt{\gamma_1}\hat{b}_{1in}[\omega]$ and $\sqrt{\gamma_2}\hat{b}_{2in}[\omega]$. We define the cavity susceptibility $\chi_c(\omega) = [\kappa/2 - i(\Delta+\omega)]^{-1}$ and a matrix $\Sigma(\omega)$ with matrix element $\Sigma_{nm}(\omega) = -i[\chi_c(\omega) - \chi^*_c(-\omega)]\, g_n g_m \alpha^* \alpha \ (n, m = 1, 2)$. Then we can obtain the following coupled-mode equations in the frequency domain

$$-i\omega \begin{pmatrix} \hat{b}_1[\omega] \\ \hat{b}_2[\omega] \end{pmatrix} = -\begin{pmatrix} \frac{\gamma_1}{2} + i\omega_1 & 0 \\ 0 & \frac{\gamma_2}{2} + i\omega_2 \end{pmatrix} \begin{pmatrix} \hat{b}_1[\omega] \\ \hat{b}_2[\omega] \end{pmatrix} - i\Sigma(\omega) \begin{pmatrix} \hat{b}_1[\omega] \\ \hat{b}_2[\omega] \end{pmatrix}. \tag{8}$$

The mechanical modes are susceptible to the drives only within their linewidths, and the mechanical modes are nearly degenerate. Therefore, the matrix $\Sigma(\omega)$ can be independent of $\omega_{1,2}$.

Consequently, Supplementary Equation (8) can be back to the two coupled-mode equations in the time domain

$$i\frac{\partial}{\partial t} \begin{pmatrix} \hat{b}_1 \\ \hat{b}_2 \end{pmatrix} = \begin{pmatrix} \omega_1 - i\frac{\gamma_1}{2} & 0 \\ 0 & \omega_2 - i\frac{\gamma_2}{2} \end{pmatrix} \begin{pmatrix} \hat{b}_1 \\ \hat{b}_2 \end{pmatrix} + \Sigma \begin{pmatrix} \hat{b}_1 \\ \hat{b}_2 \end{pmatrix}. \tag{9}$$

Here $\Sigma = \begin{pmatrix} g_1 g_1 \chi_m & g_1 g_2 \chi_m \\ g_2 g_1 \chi_m & g_2 g_2 \chi_m \end{pmatrix}$ and

$$\chi_m = \frac{P}{\hbar\omega_L} \frac{\kappa_{in}}{\frac{\kappa^2}{4} + \Delta^2} \left(\frac{(\Delta - \omega_0) + i\frac{\kappa}{2}}{(\Delta - \omega_0)^2 + \frac{\kappa^2}{4}} + \frac{(\Delta + \omega_0) - i\frac{\kappa}{2}}{(\Delta + \omega_0)^2 + \frac{\kappa^2}{4}} \right) \tag{10}$$

is the effective mechanical susceptibility. When the optomechanical coupling strengths are equal, i.e. $g_1 = g_2 = g$, the matrix Σ can be simplified as $\Sigma = \begin{pmatrix} \Lambda & \Lambda \\ \Lambda & \Lambda \end{pmatrix}$ with $\Lambda = g^2 \chi_m$. Then, the effective Hamiltonian H_{eff} can be written as

$$H_{eff}=\begin{pmatrix}\omega_1-i\frac{\gamma_1}{2}+\Lambda & \Lambda \\ \Lambda & \omega_2-i\frac{\gamma_2}{2}+\Lambda\end{pmatrix}. \quad (11)$$

The eigenfrequencies of normal modes are obtained

$$\omega_\pm=\frac{\left(\omega_1-i\frac{\gamma_1}{2}+\Lambda\right)+\left(\omega_2-i\frac{\gamma_2}{2}+\Lambda\right)}{2}\pm\frac{1}{2}\sqrt{\left[\left(\omega_1-i\frac{\gamma_1}{2}\right)-\left(\omega_2-i\frac{\gamma_2}{2}\right)\right]^2+4\Lambda^2}. \quad (12)$$

Then, we can find the critical point as $[\omega_1-\omega_2-i(\gamma_1-\gamma_2)/2]^2+4\Lambda^2=0$. When the two mechanical oscillators have the same intrinsic frequencies, the critical point of the system is $\Lambda^2-(\gamma_1-\gamma_2)^2/16=0$. When the effective coupling strength is beyond this critical point, the system enters into the strong coupling regime and the normal mode splitting appears. Please note that in the far-off red-detuned case, i.e. −Δ>>κ, the effective coupling strength is a negative real number.

Supplementary Note 2: Effective temperature and heat flux

According to the effective coupled-mode Hamiltonian (Supplementary Equation (11)), we can obtain the power spectrum density of each mechanical oscillator, i.e., $S_{\hat{b}_{1,2}^\dagger\hat{b}_{1,2}}(\omega)=\int_{-\infty}^{+\infty}\langle\hat{b}_{1,2}^\dagger(\omega)\hat{b}_{1,2}(\omega')\rangle d\omega'$, as

$$S_{\hat{b}_1^\dagger\hat{b}_1}(\omega)=\frac{\left[\left(\frac{\gamma_2}{2}\right)^2+(\omega+\delta+\Lambda)^2\right]\frac{\gamma_1k_BT_1}{\hbar\omega_1}+\Lambda^2\frac{\gamma_2k_BT_2}{\hbar\omega_2}}{\left[\frac{\gamma_1\gamma_2}{4}+\Lambda^2-(\omega+\Lambda)(\omega+\delta+\Lambda)\right]^2+\left[\frac{\gamma_2}{2}(\omega+\Lambda)+\frac{\gamma_1}{2}(\omega+\delta+\Lambda)\right]^2}, \quad (13)$$

$$S_{\hat{b}_2^\dagger\hat{b}_2}(\omega)=\frac{\left[\left(\frac{\gamma_1}{2}\right)^2+(\omega+\Lambda)^2\right]\frac{\gamma_2k_BT_2}{\hbar\omega_2}+\Lambda^2\frac{\gamma_1k_BT_1}{\hbar\omega_1}}{\left[\frac{\gamma_1\gamma_2}{4}+\Lambda^2-(\omega+\Lambda)(\omega+\delta+\Lambda)\right]^2+\left[\frac{\gamma_2}{2}(\omega+\Lambda)+\frac{\gamma_1}{2}(\omega+\delta+\Lambda)\right]^2}. \quad (14)$$

Here $\delta=\omega_2-\omega_1$ is the frequency difference between two membranes. The power spectrum density of each mechanical oscillator as a function of cavity photon number is plotted in Supplementary Figure 1. The normal mode splitting can be observed on both membranes' noise

spectra as the coupling strength is strong enough by simply increasing the cavity photon number.

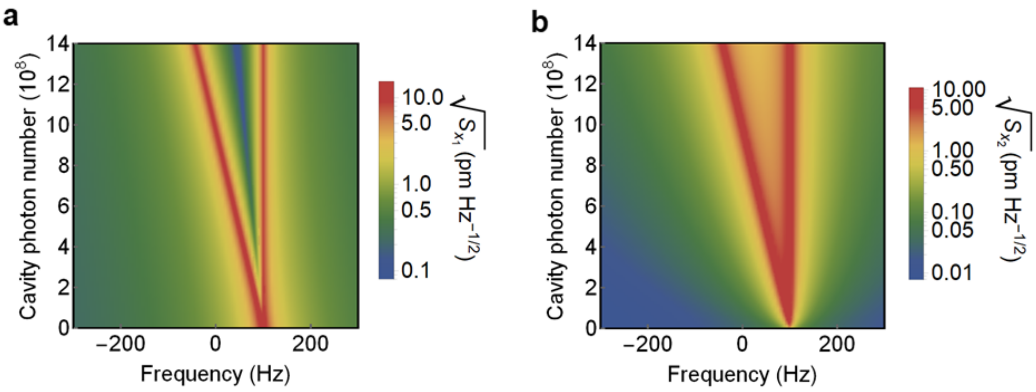

Supplementary Figure 1. Power spectral density of the mechanical motions for M1 (**a**) and M2 (**b**) as a function of cavity photon number.

The effective temperature of each mechanical oscillator in the steady states can be calculated as $T_{1,2}^{eff} = \frac{\hbar\omega_{1,2}}{k_B}\frac{1}{2\pi}\int_{-\infty}^{\infty} S_{\hat{b}_{1,2}^{\dagger}\hat{b}_{1,2}}(\omega)d\omega$. When two mechanical oscillators have the same intrinsic frequencies, i.e. $\omega_1 = \omega_2$, the effective temperatures can be formulated as

$$T_1^{eff} = T_1 + \frac{\gamma_2(T_2 - T_1)}{(\gamma_1+\gamma_2)\left(1+\frac{\gamma_1\gamma_2}{4\Lambda^2}\right)}, \tag{15}$$

$$T_2^{eff} = T_2 + \frac{\gamma_1(T_1 - T_2)}{(\gamma_1+\gamma_2)\left(1+\frac{\gamma_1\gamma_2}{4\Lambda^2}\right)}, \tag{16}$$

where $T_{1,2}$ is the temperature of thermal bath.

It is worth mentioning that the temperature defined here is based on the motions of mechanical oscillators. The motions of atoms constituting the mechanical oscillators can lead to another definition of temperature, which remain at the equilibrium values and are ignored in this work. Therefore, the heat transport arises via the optomechanical coupling due to the two non-equilibrium mechanical modes thermalized at different thermodynamic temperatures.

The net average steady heat flux from each oscillator to the its thermal bath can be calculated as $\bar{j}_{1,2} = \gamma_{1,2}k_B\left(T_{1,2}^{eff} - T_{1,2}\right)$. Therefore, the mean heat flux in steady states from the oscillator M1 to

the M2 $\left(T_1 > T_2\right)$ is [5]

$$\bar{j} = \bar{j}_2 = -\bar{j}_1 = \frac{\gamma_1\gamma_2 k_B\left(T_1 - T_2\right)}{\left(\gamma_1+\gamma_2\right)\left(1+\frac{\gamma_1\gamma_2}{4\Lambda^2}\right)}. \tag{17}$$

Now we calculate the instant heat flux. We can obtain the coupled-mode equations as

$$m\ddot{x}_1 + m\gamma_1\dot{x}_1 + m{\omega_0}^2 x_1 + m\left(2\omega_0\Lambda + 2\Lambda^2\right)\left(x_1 + x_2\right) = \eta_1, \tag{18}$$

$$m\ddot{x}_2 + m\gamma_2\dot{x}_2 + m{\omega_0}^2 x_2 + m\left(2\omega_0\Lambda + 2\Lambda^2\right)\left(x_2 + x_1\right) = \eta_2. \tag{19}$$

Here $x_{1,2} = \sqrt{\frac{\hbar}{2m\omega_{1,2}}}\left(\hat{b}_{1,2}{}^{\dagger} + \hat{b}_{1,2}\right)$ is the displacement of mechanical oscillator. η_1 and η_2 are the random thermal noise force from the environment with the statistical correlation in time as $\left\langle \eta_{1,2}\left(t\right)\eta_{1,2}\left(t'\right)\right\rangle = 2m\gamma_{1,2}k_B T_{1,2}\delta\left(t-t'\right)$. Here we have assumed that two mechanical oscillators have the same effective mass m . The instant heat flux between the mechanical oscillator and the thermal bath can be defined as the negative instant power done by the environment random force and the dissipation to the bath, which is

$$j_{1,2} = -\left(-m\gamma_{1,2}\dot{x}_{1,2} + \eta_{1,2}\right)\dot{x}_{1,2}. \tag{20}$$

By using Supplementary Equations (18) and (19), Supplementary Equation (20) can be rewritten as

$$j_1 = -\left[m\ddot{x}_1 + m{\omega_0}^2 x_1 + m\left(2\omega_0\Lambda + 2\Lambda^2\right)\left(x_1 + x_2\right)\right]\dot{x}_1, \tag{21}$$

$$j_2 = -\left[m\ddot{x}_2 + m{\omega_0}^2 x_2 + m\left(2\omega_0\Lambda + 2\Lambda^2\right)\left(x_2 + x_1\right)\right]\dot{x}_2. \tag{22}$$

Therefore, the instant heat flux j from the oscillator M1 to the M2 $\left(T_1 > T_2\right)$ is formulated as [6,7]

$$j = -m\left(2\omega_0\Lambda + 2\Lambda^2\right)x_1\dot{x}_2. \tag{23}$$

Supplementary Note 3: Experimental details

The detailed experimental setup is shown in Supplementary Figure 2, which is similar to the one used in Supplementary Reference [4]. A 795 nm diode laser (Laser 1) is split into two beams

by using PBS1. The weak locking beam passes through an EOM and is used to stabilize the experimental cavity via the Pound-Drever-Hall technique. The other strong beam (the pumping beam) passes through the AOM for controlling the frequency detuning between the pumping beam and the experimental cavity system. Laser 1 is stabilized to an ultrastable optical cavity for the purpose of linewidth narrowing, which is not shown in Supplementary Figure 2.

The experimental cavity system is a two-membrane-in-the-middle cavity optomechanical system, where two flexible stoichiometric silicon nitride (SiN) membranes are placed inside an optical Fabry-Perot cavity. The optical cavity consists of two identical flat concave laser mirrors represented by CM1 and CM2 in Supplementary Figure 2. The membranes have a thickness of 50 nm and a 1×1 mm^2 size. We can parametrically control the position and the natural frequency of each membrane separately with two PZTs attached to every membrane. The mirrors and membranes are mounted on a breadboard separately. The whole two-membrane-in-the-middle cavity optomechanical system is placed inside a vacuum chamber with a pressure better than 10^{-7} torr by using an ion pump.

In order to measure the oscillation of each membrane individually, a 1064 nm Nd:YAG laser (Laser 2) is used and split into two weak probe beams. Each probe beam passes through the cavity mirror and the reflection from the membrane is monitored by the corresponding photodetectors (PD1 and PD2), as shown in Supplementary Figure 2. The cavity mirrors have high reflectivity at 795 nm but low reflectivity at 1064 nm. The detected signal from PD1 (PD2) is split into two ways that are sent into a spectrum analyzer for the power spectrum measurements and a lock-in amplifier for the real-time dynamic measurements of the mechanical motions, respectively. In experiment, the high temperature of the mechanical oscillator (M1) can be obtained through adding white noise voltage on the PZT.

The damping rates of the membranes, γ_1 and γ_2, are measured by the mechanical ring-down approach [8]. To measure the optomechanical coupling strengths, g_1 and g_2, according the definition, i.e., $g=-\frac{\partial\omega_c}{\partial x}\sqrt{\frac{\hbar}{2m\omega_0}}$, we need to know the derivative of the cavity resonance frequency to the displacement of membrane. We keep one membrane position fixed, and change the position of the other membrane through controlling the voltage on the piezo. Then we can obtain the cavity resonance shift by recording the cavity transmission spectrum [4]. Therefore, we

can keep the optomechanical coupling strengths equal, i.e. $g_1 = g_2$, during the measurements.

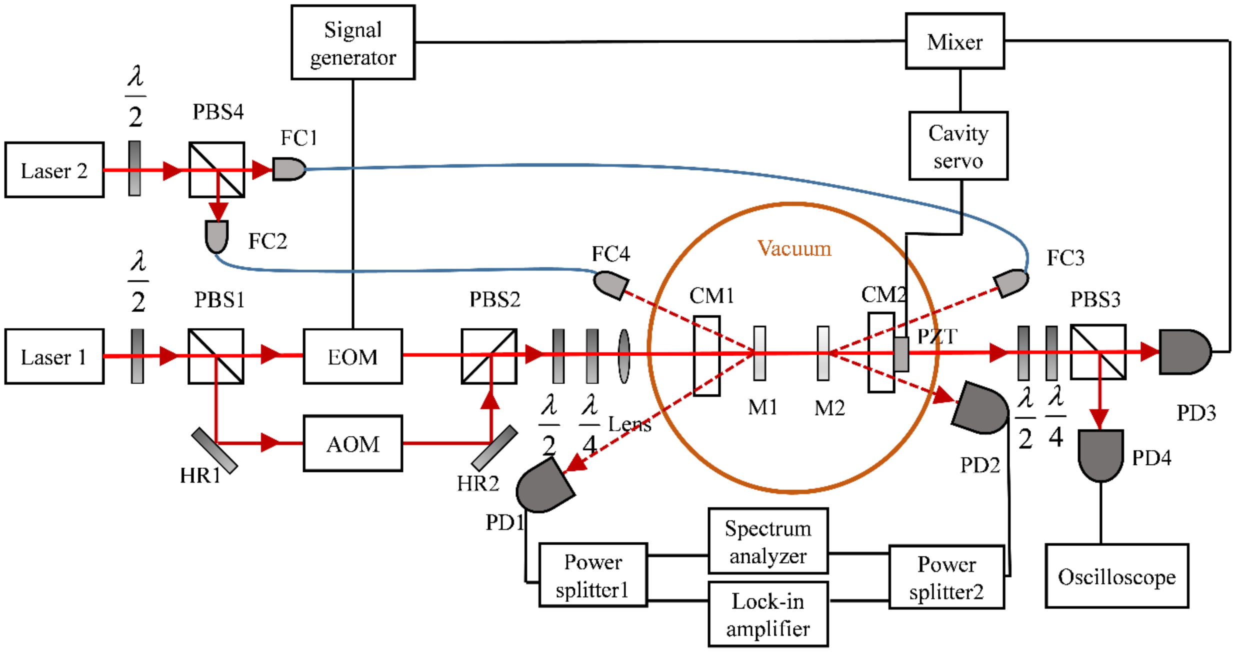

Supplementary Figure 2. Experimental setup. PBS, polarization beam splitter; FC, fiber coupler; HR, high reflection mirror; AOM, acousto-optic modulator; EOM, electro-optic modulator; M, stoichiometric silicon nitride membrane; CM, cavity mirror; PD, photodetector; λ/2, half-wave plate; λ/4, quarter-wave plate; PZT, piezoelectric transducer.

The instant heat flux is obtained based on the measurements with the lock-in amplifier. The time derivative of the displacement is $\dot{x}_i(t) = \left(\dot{X}_i - \omega_r Y_i\right)\cos\omega_r t - \left(\dot{Y}_i + \omega_r X_i\right)\sin\omega_r t$. Here ω_r is the reference frequency, and $X_i(t)$ and $Y_i(t)$ are the quadrature components, which can be direction obtained from the lock-in amplifier. According to Supplementary Equation (23), the instant heat flux j can be rewritten as

$$\begin{aligned} j = -2m(\omega_0\Lambda + \Lambda^2)[X_1(\dot{X}_2 - \omega_r Y_2)\cos^2\omega_r t + Y_1(\dot{Y}_2 + \omega_r X_2)\sin^2\omega_r t \\ -(X_1(\dot{Y}_2 + \omega_r X_2) + Y_1(\dot{X}_2 - \omega_r Y_2))\cos\omega_r t\sin\omega_r t]. \end{aligned} \tag{24}$$

The integration time of the lock-in amplifier is at the order of 100 μs, which is much larger than the period of the oscillation. Therefore, the instant heat flux j can be presented as

$$j = -m\left(\omega_0\Lambda + \Lambda^2\right)\left[X_1\left(\dot{X}_2 - \omega_r Y_2\right) + Y_1\left(\dot{Y}_2 + \omega_r X_2\right)\right]. \tag{25}$$

In the strong coupling regime, the noise spectrum shows two peaks. The eigenmodes are (1,1) and (1,-1) under the condition that $\omega_1 \approx \omega_2$ and $\gamma_1 \approx \gamma_2$, which are called the center-of-mass mode and breathing mode, respectively. To experimentally verify the collective motions of membranes, we use lock-in amplifier. By choosing the reference frequency of lock-in at the peak of each normal mode, and select the lowpass filter ~10 Hz, we find the motions of two membranes are indeed in-phase and out-of-phase for two normal modes, respectively, which are shown in Supplementary Figure 3.

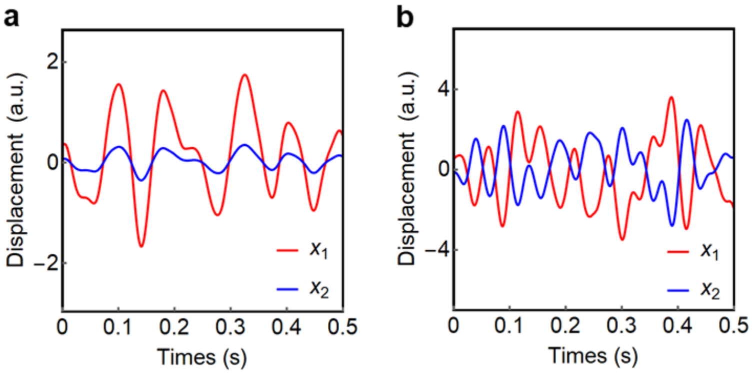

Supplementary Figure 3. Motions of membranes in the strong coupling regime. **a,b** The center-of-mass and breathing modes, respectively.

Supplementary Note 4: Calibration of the effective temperatures

The thermal motion of each membrane oscillator is measured by the 1064 nm laser. The signal-to-noise ratio of the thermal noise spectrum under room temperature is better than 15 dB. The displacement signal of each membrane from the photoelectric detector is divided into two ways. One enters into the spectrum analyzer, the other goes in the lock-in amplifier. We can do the calibrations of the effective temperature by using data either from the spectrum analyzer or the lock-in amplifier, and the obtained results are consistent with each other.

According to the equipartition theorem, i.e., $k_B T_i^{eff} = m\omega_i^2 \langle x_i^2 \rangle$, the calibration for the effective temperature of each mechanical mode is based on the thermal noise of the fundamental mode at room temperature. By using the data of spectrum analyzer, the effective temperature of each mechanical mode can be obtained from the area of the power spectrum of the thermal motion in the frequency domain. By using the data of lock-in amplifier, the mechanical displacement of

each oscillator can be decomposed into $x_i(t) = X_i(t)\cos\omega_r t - Y_i(t)\sin\omega_r t$, where ω_r is the reference frequency, and $X_i(t)$ and $Y_i(t)$ are the quadrature components. Thus, the effective temperature can be obtained according to $T_i^{eff} = m\omega_i^2 \langle X_i^2 + Y_i^2 \rangle / 2k_B$. Hence, we can directly obtain the effective temperature with $T_{High}^{eff} = T_{Room}^{eff} \langle X_{High}{}^2 + Y_{High}{}^2 \rangle / \langle X_{Room}{}^2 + Y_{Room}{}^2 \rangle$. Here T_{High}^{eff} and T_{Room}^{eff} are the effective temperatures for the cases of high temperature and room temperature, respectively. Figure S4 shows the lock-in data at three different effective temperatures and the corresponding probability density distribution, which obeys the Boltzmann distribution.

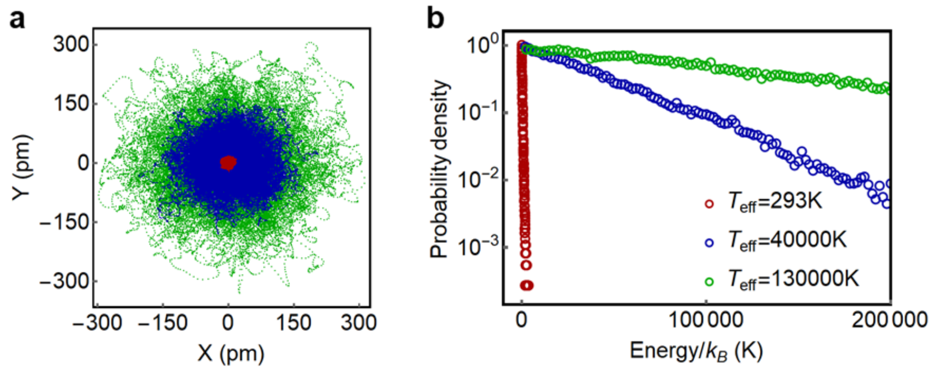

Supplementary Figure 4. Effective temperatures. **a,** The quadrature components of lock-in measurements at three different effective temperatures. **b,** The probability density distributions at different effective temperatures, which obey the Boltzmann distribution $P(E) \propto e^{-E/k_B T_{eff}}$.

The effective temperature can also be calibrated by using the spectrum analyzer. The noise power spectra at room temperature and high temperature (with additional white noise driving) are shown in Supplementary Figures 5a and 5b, respectively. The integrated areas for Supplementary Figures 5a and 5b are obtained approximately as 67 and 3×10^4 in arbitrary units but in the same procedure, respectively. The room temperature is ~ 300 K. Therefore, we can obtain that the effective temperature of the membrane in Supplementary Figure 5b is ~ 130000 K. It is worthy to mentioning that the root-mean-square displacement of membrane, $x_{rms} = \sqrt{\langle x^2 \rangle}$, at room temperature and high temperature (130000 K) are 4.5 pm and 94 pm, respectively.

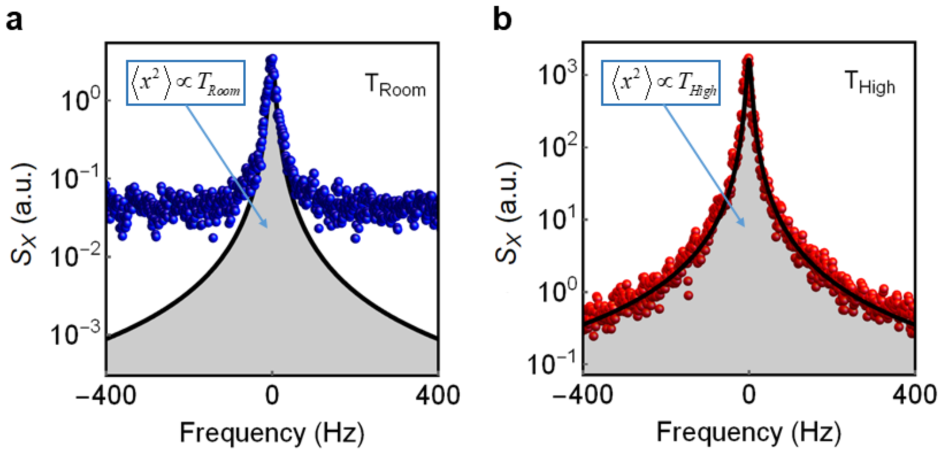

Supplementary Figure 5. Power spectral density. **a,b,** The cases of room temperature and high temperature, respectively. The blue and red dots are the experimental data. The black curves are the fitting. The grey shadows are the areas for the integration.

Supplementary Note 5: Instant heat flux in the weak coupling regime

The time evolution of instant heat flux in the weak coupling regime is plotted in Supplementary Figure 6, in compassion with the strong coupling case shown in Fig. 3 in the main text. Although the heat flux traces have completely different behaviors in the strong and weak coupling regimes, they have similar probability density function, both having exponential tails and positive mean values. The asymmetry of the probability density function in the weak coupling case is due to the large difference of the effective temperatures of two membranes.

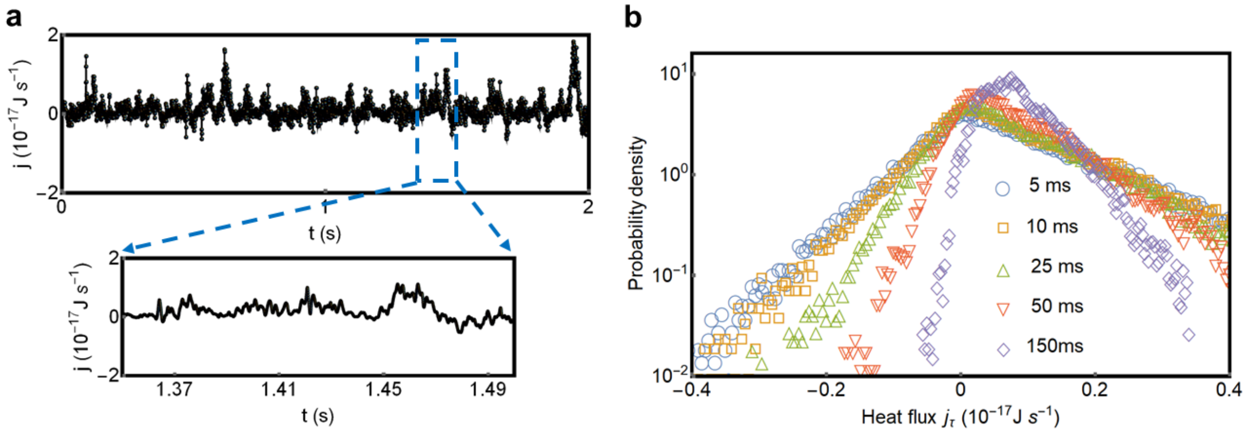

Supplementary Figure 6. Instant heat flux in the weak coupling regime. **a,** Time evolution of instant heat flux. The inset is the detailed instant heat flux for the purpose of clarity. **b,** The

corresponding probability density functions at different integration time.

Supplementary References

[1] Markus Aspelmeyer, Tobias J. Kippenberg, and Florian Marquardt, Cavity optomechanics, Rev. Mod. Phys. **86**, 1391 (2014).

[2] H. Xu, D. Mason, L. Jiang, and J. G. E. Harris, Topological energy transfer in an optomechanical system with an exceptional point, Nature **537**, 80-83 (2016).

[3] F. Bemani, A. Motazedifard, R. Roknizadeh, M. H. Naderi, and D. Vitali, Synchronization dynamics of two nanomechanical membranes within a Fabry-Perot cavity, Phys. Rev. A **96**, 023805 (2017).

[4] Jiteng Sheng, Xinrui Wei, Cheng Yang, and Haibin Wu, Self-organized synchronization of phonon lasers, Phys. Rev. Lett. **124**, 053604 (2020).

[5] King Yan Fong, Hao-Kun Li, Rongkuo Zhao, Sui Yang, Yuan Wang, and Xiang Zhang, Phonon heat transfer across a vacuum through quantum fluctuations, Nature **576**, 243–247 (2019).

[6] Gabriel Barton, Classical van der Waals heat flow between oscillators and between half-spaces, J. Phys.: Condens. Matter **27**, 214005 (2015).

[7] A. Bérut, A. Imparato, A. Petrosyan, and S. Ciliberto, Stationary and transient fluctuation theorems for effective heat fluxes between hydrodynamically coupled particles in optical traps, Phys. Rev. Lett. **116**, 068301 (2016).

[8] Shuhui Wu, Jiteng Sheng, Xiaotian Zhang, Yuelong Wu, and Haibin Wu, Parametric excitation of a SiN membrane via piezoelectricity, AIP Advances **8**, 015209 (2018).
